# Industrialization’s eye view on theranostic nanomedicine

**DOI:** 10.3389/fchem.2022.918715

**Published:** 2022-08-19

**Authors:** Maharajan Sivasubramanian, Li-Jie Lin, Yu-Chao Wang, Chung-Shi Yang, Leu-Wei Lo

**Affiliations:** Institute of Biomedical Engineering and Nanomedicine, National Health Research Institutes, Zhunan, Taiwan

**Keywords:** translational nanomedicine, drug nanoformulation, theranostics, multifunctional nanotheranostics, multistep nanotheranostics, ISO and ASTM international, investigational new drug (IND)

## Abstract

The emergence of nanomedicines (NMs) in the healthcare industry will bring about groundbreaking improvements to the current therapeutic and diagnostic scenario. However, only a few NMs have been developed into clinical applications due to a lack of regulatory experience with them. In this article, we introduce the types of NM that have the potential for clinical translation, including theranostics, multistep NMs, multitherapy NMs, and nanoclusters. We then present the clinical translational challenges associated with NM from the pharmaceutical industry’s perspective, such as NMs’ intrinsic physiochemical properties, safety, scale-up, lack of regulatory experience and standard characterization methods, and cost-effectiveness compared with their traditional counterparts. Overall, NMs face a difficult task to overcome these challenges for their transition from bench to clinical use.

## Introduction

Nanomedicine (NM) is a term assigned to nanoparticles (NPs) that are employed in biomedical applications such as biosensing, diagnostic imaging, and therapy. In their infancy, NPs were used to improve the solubility and stability of poorly water-soluble drugs (i.e., nanoformulation) and restrict their sporadic distribution, thus minimizing off-target toxicity (Rosenblum et al., 2018; Mitchell et al., 2021; Shah et al., 2021; Vargason et al., 2021). A classic example of this is Doxil™, a liposomal doxorubicin (Dox) formulation approved for clinical applications by the United States’ Food and Drug Administration (FDA) in 1995 (Gabizon et al., 2003; Robert et al., 2004; Barenholz, 2012). Doxil™ demonstrated a distinctive distribution pattern compared with that of free Dox. In addition, patients who received this nanoformulation showed a 300-fold increase in the area under the curve (AUC) compared with the AUC of those who were administered free Dox (Gabizon et al., 2003). Following this success, rigorous advancements in materials science and nanotechnology have been achieved, over several decades. Sophisticated NPs have been synthesized to perform a variety of important functions, rather than serving solely as a drug carrier. Modern NPs can execute both diagnostic and therapeutic functions (referred to as theranostics) (Zou et al., 2016; Cheng X. et al., 2021). NPs *per se* can perform therapeutic functions, such as acting as a radiosensitizer by attenuating high-energy X-rays to destroy pathological tissue, or absorb light of suitable wavelength to generate cytotoxic reactive oxygen species (ROS) or photothermal heat (Liu R. et al., 2019; Luo et al., 2020; Escudero et al., 2021; Xie et al., 2021). Indeed, the ability to distinguish pathological tissue from normal tissue by harnessing distinct interactions and processing pathways is an important property of NPs.

NPs have a number of advantages compared with small molecule drugs: 1) the high surface area-to-volume ratio of nanomaterials enables them to load drugs, which significantly alters their pharmacokinetics (PK); 2) enhanced drug accumulation at the intended pathological tissue can be achieved either by attaching targeting molecules (active targeting mechanism) (Danhier et al., 2010; Bazak et al., 2015; Kue et al., 2016) or through an enhanced permeability and retention (EPR) effect (Maeda et al., 2000) (passive targeting mechanism), avoiding non-specific distribution and related toxicity; 3) NPs can increase the water solubility of poorly water-soluble drugs and thus their biocompatibility; (Tran and Tran, 2019); 4) NPs impart multifunctional features, such as simultaneous diagnostic imaging and therapy (i.e., theranostics) (Chuang et al., 2020), and 5) all of these features collectively contribute toward the development of personalized medicine.

Theranostic NPs can provide vast quantities of information to clinicians for patient stratification, treatment planning, and important factors that influence the disease environment, which may lead to optimal personalized medicine. Multi-therapy NPs have also been developed that induce a robust therapeutic response by complementing each other. For instance, for the radio-photothermal ablation of tumors, a well-studied combination therapy platform has been established (Zhang et al., 2020). Photothermal therapy (PTT) is executed first, because when tumors are heated up to 43°C, tumor blood flow can be increased several fold (Song et al., 2005; Elming et al., 2019). This not only promotes increased vascular permeability to macromolecules (Song et al., 2001; Lee et al., 2018) but also enriches the tumor with oxygen. As a consequence, the level of hypoxia in the tumor will be diminished, resulting in favorable radiotherapeutic effects.

Another interesting approach is the step-by-step or multi-step performance of NPs. Such steps may include reaching the disease location, followed by penetrating the disease core, and finally delivering the therapeutics. For instance, stimuli-responsive dendritic polymer NPs of 100-nm diameter were developed that could reach a tumor’s location. In the acidic tumor microenvironment (TME), pro-drug-tagged small dendritic NPs were released and were able to penetrate the tumor core due to their size. The pro-drug was finally released intracellularly and executed its therapeutic effects (Li et al., 2016). Although these developments are encouraging, modern NPs face several challenges for their translation into clinical settings.

Tables 1, 2 show NMs that are already on the market or undergoing clinical trials. Only a few multifunctional NMs have entered clinical trials, however, due to the complicated nanocarrier design of multifunctional NMs. Therefore, from a clinical perspective, relatively simple designs of drug nanoformulation are needed to meet the needs of clinical applications. In this article, we first discuss multifunctional NMs that have been investigated in the extant literature. Second, we present some of the clinical challenges presented by multifunctional nanocarriers. We explore the impact of these challenges on the gap between clinical development and academic research. Although numerous challenges exist to gain clinical approval, the necessity of multifunctional nanocarriers in treating certain diseases is demonstrable.

**TABLE 1:** Examples of FDA-approved nanomedicines.

Nanoplatform	Trade name	Application	Year approved
Liposome	Vyxeos®	Acute myeloid leukemia	2017
Liposome	Onivyde®	Metastatic pancreatic cancer	2015
Liposome	Doxil®	Kaposi’s sarcoma	1995
Polymer (PEG)	Mircera®	Chronic renal disease for pediatric patients	2018
Polymer (PEG)	Adynovate®	Hemophilia	2015
Polymer (PEG)	Rebinyn®	Hemophilia	2017
Nanocrystals	NanOss®	Bone graft substitute	2014
Nanocrystals	Ryanodex®	Skeletal muscle relaxant in malignant hyperthermia	2014
Nanocrystals	Abilify Maintena®	Schizophrenia	2013
Lipid NPs	Onpattro®	hATTR	2018
Lipid NPs	Moderna COVID-19 Vaccine®	COVID-19 vaccine	2020
Lipid NPs	Comirnaty®	COVID-19 vaccine	2020

**TABLE 2:** Nanomedicines undergoing clinical trials.

Nanoplatform	Company	Application	National clinical trial number (NCT)
Liposome Topotecan (TL1)	Spectrum Pharmaceuticals	Small lung, ovarian, and other advanced tumors	NCT00765973
Liposomal Annamycin	Moleculin Biotech	Leukemia	NCT03315039
PEGylated liposomal cisplatin (LiPlaCis)	Oncology Venture	Advanced or refractory tumors	NCT01861496
Transferrin ligand-directed liposomal oxaliplatin (MBP-426)	Mebiopharm	Gastric, gastroesophageal, or esophageal adenocarcinoma	NCT00964080
Anti-EGFR-targeting liposomal doxorubicin (C225-ILs-dox)	University Hospital, Basel, Switzerland	Gliomas	NCT03603379
Thermally sensitive liposomal doxorubicin (ThermoDox)	Celsion	Breast cancer	NCT03749850
Liposomal small activating RNA of CEBPA (MTL-CEBPA)	MiNA Therapeutics	Liver cancer	NCT02716012
Liposomal p53 plasmid (SGT-53)	SynerGene Therapeutics	CNS malignancies	NCT03554707
Liposomal EphA2 siRNA (EphA2-siRNADOPC)	M.D. Anderson *Cancer* Center	Advanced tumors	NCT01591356
Liposomal DNA complex containing immunostimulatory CpG and non-CpG motifs	Colby Pharmaceutical	Leukemia	NCT00860522
Albumin-bound rapamycin nanoparticle (ABI-009)	Aadi Bioscience	Solid tumors	NCT02975882
		Non-adipocytic soft tissue sarcomas	NCT03660930
		Colorectal cancer	NCT03439462
		Bladder cancer	NCT02009332
		Advanced sarcoma	NCT03190174
		Myeloma ongoing	NCT03657420
Hafnium-oxide nanoparticle (NBTXR3)	Nanobiotix	Head and neck cancer or non-small-cell lung cancer	NCT03589339
		Prostate adenocarcinoma	NCT02805894
		Liver cancer	NCT02721056
Camptothecin−cyclodextrin conjugate (CRLX101)	NewLink Genetics	Small-cell lung cancer	NCT02769962
		Solid tumors	NCT02648711
Liposomal survivin-based synthetic peptide antigens and an adjuvant (DPX-Survivac)	Immuno Vaccine	Ovarian cancer lymphoma	NCT03029403
			NCT02323230
			NCT03349450
Albumin-bound rapamycin nanoparticle (ABI-009)	Aadi Bioscience	Glioblastoma, perivascular epithelioid cell tumor, lung or gastroenteropancreatic cancer	NCT03463265
			NCT02494570
			NCT03670030

## Tailored design—Multifunctional nanomedicine

Drug nanoformulations have been under development for several decades. Currently, certain challenges have been identified in the treatment of diseases, such as the inefficient treatment of certain diseases when using a single drug-carrier, or the inability to track nanocarriers to provide precision therapy. Multifunctional NMs, which can perform two or more functions, can overcome these obstacles in the treatment of diseases. In this review article, and according to the substances encapsulated in the drug carrier, multifunctional NMs are divided into theranostics and multitherapy NPs. NPs that possess the capability to both deliver a therapeutic agent and yield diagnostic information are termed theranostics. On the other hand, two or more different functional therapeutics that are combined into one vehicle form what are known as multitherapy NPs. The therapeutic difference between combinational drug therapies and multifunctional NPs may arise due to the following reasons. In the former approach, fixing the dosage ratio of two drugs is challenging, due to abnormal pharmacokinetics (PK), increased toxicity due to non-specific distribution, inappropriate combinations of drugs that might result in modest therapeutic efficacy, and an inability to provide an accurate diagnosis. However, in the latter approach, these concerns could be avoided because of the following reasons. Many NPs *per se* possess therapeutic capabilities, non-specific distribution could be prevented by attaching targeting molecules to NPs thus reducing toxicity, and single entity NPs can perform multiple diagnostic and therapeutic functions. In this section we will discuss the different types of multifunctional NMs.

## Theranostic nanoparticles

Precision medicine (PM) is a medical model that tailors treatments for individual patients. The ability to simultaneously treat patients and monitor the targeting of treatment by using theranostic NPs can provide critical feedback to physicians and researchers, such as whether a tumor’s failure to respond arises from drug resistance or insufficient drug delivery (Bikram et al., 2007; Cheon and Lee, 2008; Yavuz et al., 2009; Lee et al., 2011; Xiao et al., 2012). Therefore, theranostics, i.e., the combination of therapeutic and diagnostic capability in a single system, may realize the promise of PM.

There are some inorganic materials, which have therapeutic or diagnostic capabilities, that can be used as a platform to encapsulate diagnostic or therapeutic agents, respectively. Sun et al*.* designed ^64^Cu-integrated gold nanorods modified with arginine-glycine-aspartate (RGD) peptide for positron emission tomography (PET) image-guided PTT. In whole-body PET imaging of mice, RGD-^64^CuAuNR808 showed a high degree of tumor-targeting ability compared with non-targeting or pre-treated RGD NPs. RGD-^64^CuAuNR808 with laser irradiation exhibited obvious inhibition of the tumor growth path compared with laser irradiation only (Sun et al., 2014). Qi et al. developed *in situ* biomineralized manganese carbonate (BMC) NPs using poly (ethylene glycol)-b-poly (l-aspartic acid) as a template, and the resultant nanoplatform served as a tumor-specific theranostic system. Mineralized NPs decomposed to release Mn^2+^ ions and simultaneously generated CO_2_ gas, showing functions as a TME-responsive dual magnetic resonance (MR)/ultrasound (US) imaging agent. Time-dependent analysis of MR imaging after intravenous administration of BMC NPs revealed a gradual T1 signal increase in tumors. T1 signal intensity reached a maximum at 8 h post-injection and decreased at 24 h. Strong US signals were observed in the tumor 10 min post i.t. administration of BMC, and the signal intensity continued to increase throughout the entire study (120 min). When loaded with Dox, BMC-Dox exhibited a high degree of tumor inhibition effects due to the cooperative therapeutic functions of Dox and the chemodynamic performance of Mn^2+^ (Qi et al., 2021).

**FIGURE 1:**
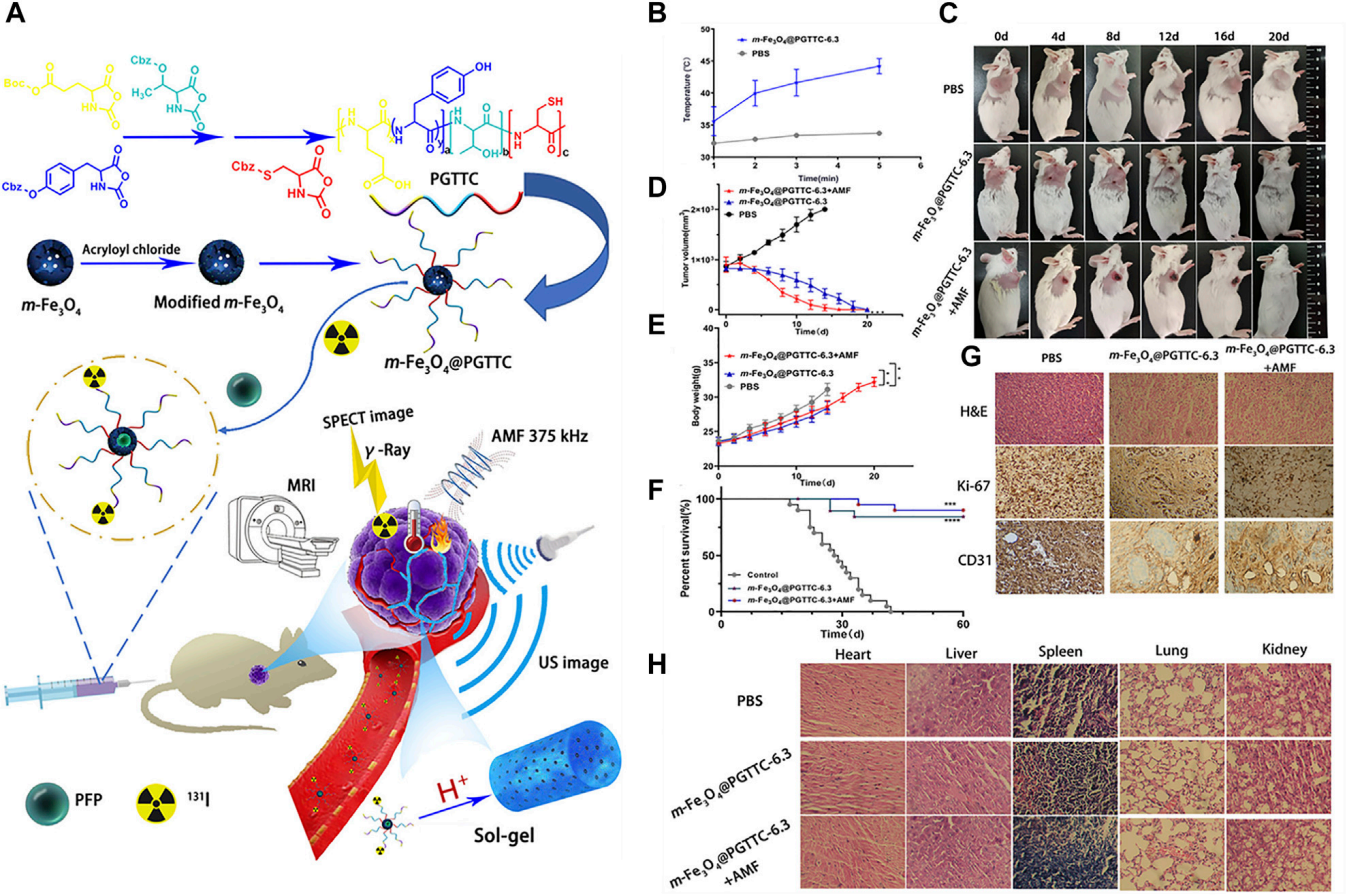
**(A)** Design and application of PFP-m-Fe3O4@PGTTC, **(B)** temperatures of the tumor tissue at various time points, **(C)** photographs of H22-tumor-bearing mice with various treatments. **(D)** Comparison of subcutaneous tumor volume changes among groups, **(E)** comparison of subcutaneous body weight changes among groups, **(F)** survival curves of mice within 60 days, **(G)** representative H&E-, Ki-67-, and CD31-stained images of tumor slices collected from mice in various groups, and **(H)** H&E-stained images of the major organs (heart, liver, spleen, lung, and kidney) from various groups. ****p < 0.0001, ***p < 0.001, and **p < 0.01. Reproduced with permission (Lu et al., 2021). Copyright 2021, American Chemical Society.

Lu et al*.* developed a simple theranostic system for non-interventional target-embolization therapy (NTE). The nanosystem comprises perfluoropentane mesoporous Fe_3_O_4_ coated with triblock-polypeptide (PFP-m-Fe_3_O_4_@PGTTCs) that integrates magnetic hypothermia, US imaging, MR imaging, and NTE. When intravenously administered in mice or rabbits bearing tumors, PFP-m-Fe_3_O_4_@PGTTCs preferentially accumulated in the tumor and underwent *in situ* gelation due to the coated biodegradable and biocompatible PGTTCs (Figure 1). As a consequence, tumor blood vessels were occluded, and catheter-free NTE was realized. In the presence of an alternating magnetic field, PFP-m-Fe_3_O_4_ generated a magnetocaloric effect that demonstrated therapeutic effects, and MR and US imaging were also accomplished (Lu et al., 2021). Theranostic Fe^3+^ coordinated croconaine encapsulated with bovine serum albumin (Cro-Fe@BSA) for combined ferroptosis and PTT was developed by Zeng et al. When administered in a subcutaneous tumor-bearing mouse model, the nanomedicine accumulated in the tumor, followed by a cascade of reactions. First, the high levels of glutathione and low pH of the TME reduced Fe^3+^ to Fe^2+^ ions, which detached the croconaine molecules while retaining their photothermal properties. When irradiated with a laser, their photoacoustic (PA) imaging and photothermal properties were initiated. In addition, the PTT effect could amplify the Fenton reaction to form hydroxyl radicals and a labile iron pool. These cooperative effects constituted MR and PA imaging, ferroptosis, and PTT for cancer theranostics (Zeng et al., 2022). Chuang et al*.* developed and used Y_2_O_3_:Eu@SiO_2_ as a nanoscintillator for X-ray activated photodynamic therapy (PDT) in deep cancer theranostics. The annealed Y_2_O_3_:Eu@SiO_2_ demonstrated superior PDT effects when irradiated with X-rays, by generating cytotoxic ROS in the absence of photosensitizers (PS). In a tumor-bearing mouse model, i.t. administered Y_2_O_3_:Eu@SiO_2_ in the presence of X-rays not only induced strong tumor-inhibiting effects but also exhibited radioluminescence, which increased with increasing nanoparticle concentration. In addition, *in vivo* PA imaging showed redistribution of oxygen saturation and re-oxygenation in hypoxic tumors (Chuang et al., 2020).

## Multitherapy nanoparticles

Due to the defense mechanism of tumors and the different stage requirements of therapy, single-therapy NPs cannot effectively achieve the treatment of disease. Multitherapy NPs, however, which contain two or more therapeutics, can realize the effective treatment of disease. In this section we will review several examples of multitherapy NPs. Tumor neovascularization and cancer cell proliferation are characteristics of cancer disease. Cancer cell proliferation involves DNA replication, tubulin polymerization, and the inhibition of apoptosis. Furthermore, tumors require neovascularization to provide tumor cells with nutriments and oxygen. A combination of antivascular drugs and cytotoxic agents may solve the problem of inefficient treatment when using a single anticancer drug, either antimitotic or anti-angiogenic (Escorcia et al., 2010; Rosenzweig, 2012; Johannessen et al., 2013). Researchers investigated self-assembled NPs (SQ-gem/isoCA-4 NAs) that contained the anticancer drug gemcitabine conjugated with squalene (SQ-gem) together with isocombretastatin A-4 (isoCA-4), which is a vasculature disruptor. Tumor growth inhibition was significantly improved following treatment with SQ-gem/isoCA-4 NAs compared with free gemcitabine and isoCA-4 (Maksimenko et al., 2014).

PTT and PDT are two different types of phototherapy. In PTT, the PS converts energy into heat to kill cancer cells under light irradiation. On the other hand, the PS of PDT transfers energy from light to molecular oxygen, to generate ROS and elicit cell death (Li et al., 2020). A combination of phototherapy and chemotherapy drugs can achieve more effective therapy. Researchers designed a temperature-responsive upconversion nanosystem (TR-UCNS), which contained a photothermal agent (PdPc) and a thermally responsive drug-release unit (DPPC micelles). The sequence involving chemotherapy first, followed by PTT, achieved higher therapeutic efficacy compared with the sequence of PTT followed by chemotherapy or single therapy *in vitro*; it also significantly inhibited tumor growth compared with simultaneous treatment with chemotherapy and PTT (Zhu et al., 2018). All-in-one hollow silica NPs encapsulating Dox, chlorin e6 (Ce6), and Mn ions (aHNF) through a complexation effect and molecular weight selection were developed by Yan et al*.* for chemotherapy and MR-guided PDT to treat hepatocellular carcinoma. When intravenously administered, aHNF selectively accumulated in the tumor through the EPR effect and released Dox and Ce6 along with Mn ions. Laser irradiation of tumors resulted in PDT effects, while the co-delivery of Mn ions elevated the cytotoxicity of chemoPDT by elevating intracellular oxidative stress and destabilizing metabolic homeostasis. The released Mn ions also provided MR imaging to guide the PDT (Yan et al., 2022).

**FIGURE 2:**
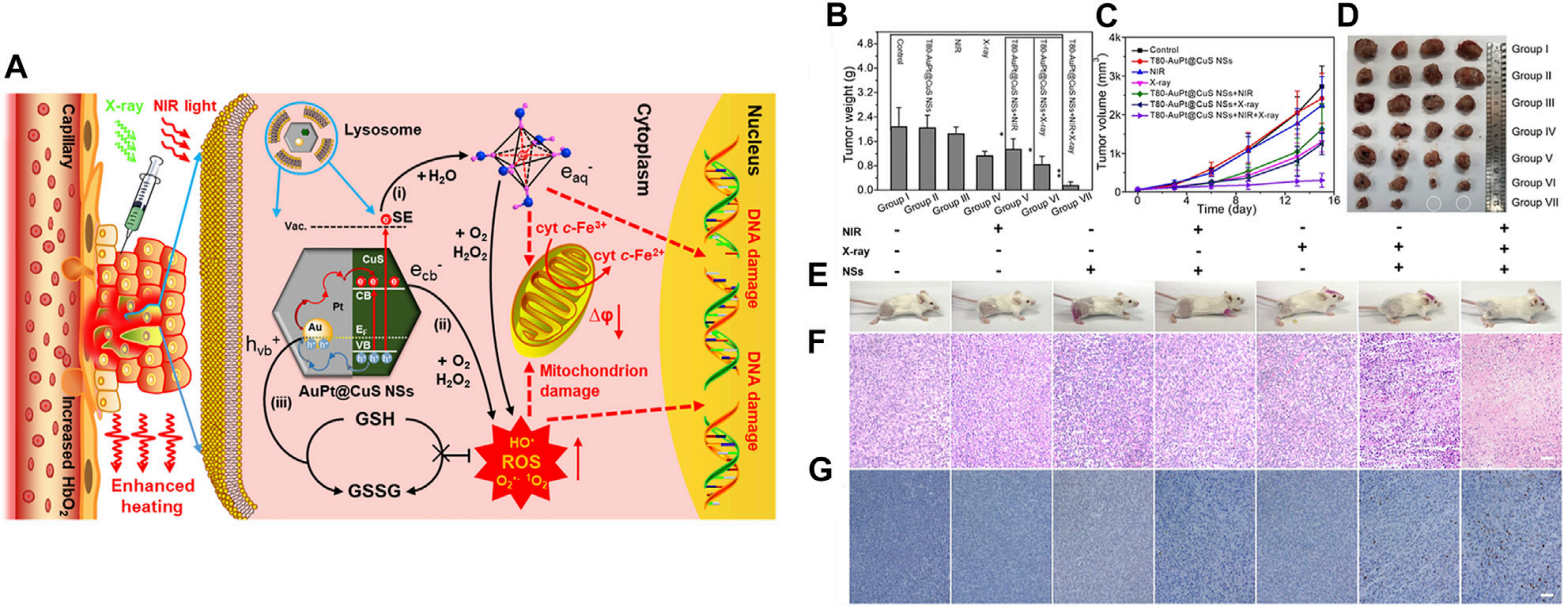
**(A)** Schematic representation of AuPt@CuS NSs for enhanced synergistic radio-photothermal therapy, **(B)** weights of dissected tumors from each group, **(C)** relative tumor volumes after various treatments (*n* = 4), **(D)** digital photographs of the dissected tumors, **(E)** photographs of mice after treatment. **(F,G)** H&E-stained images of tumor sections following treatment. Reproduced with permission (Cai et al., 2021). Copyright 2021, American Chemical Society.

Xiang et al*.* fabricated silica-coated bismuth NPs covered with lauric acid that contained a prodrug (BSBCL) for radio-photothermal therapy (Xiang et al., 2021). The Si coating imparted hydrolytic stability and photothermal and radiosensitizing properties of the Bi NPs, while the lauric acid acted as a temperature-sensitive gatekeeper. In a tumor-bearing mouse model, when illuminated with near-infrared (NIR) light, i.t. administered BSBCL increased the temperature that lauric acid melts at, releasing the prodrug. The high level of H_2_O_2_ in a tumor activated the prodrug, resulting in irreversible depletion of glutathione, which is a free radical scavenger. The heat generated also increased the blood flow in tumors, thereby increasing oxygen saturation in the tumors and relieving hypoxia. Oxygen-enriched tumors, when irradiated with X-rays, produced copious quantities of ROS due to the radiosensitizing properties of bismuth NPs. Due to these combined actions, strong tumor inhibition was observed compared with that seen in the controls. Cai et al*.* developed plasmonic heterostructures by coating AuPt NPs over CuS nanosheets, which endowed them with theranostic ability for dual PA/computed tomography imaging and radio-phototherapy (Figure 2). Tween80-modified plasmonic structures (T80-AuPt@CuS) achieved good stability and photothermal conversion efficiency due to electromagnetic enhancement at the heterojunctions. In a tumor-bearing mouse model, i.t. injected T80-AuPt@CuS increased blood flow due to NIR-mediated heating. The resultant increase in tumor oxygen saturation was measured by PA imaging. Synergistic radio-phototherapy in a tumor mouse model achieved a tumor inhibition rate of 92.8%. This enhanced therapeutic efficacy might have been due to tumor re-oxygenation by PTT followed by amplified ROS production by radiotherapy (RT) (Cai et al., 2021).

Following a spinal cord injury (SCI), patients must immediately be injected with glial cell line-derived neurotrophic factor (GDNF) in the injured area to prevent the nerves from being permanently damaged. Then, the formation of glial scars must be prevented by using drugs that scavenge scars, to repair the nerves. In the case of SCI, two different drugs must be administrated at different times. Yang and co-workers designed biodegradable PLGA NP-encapsulated GDNF that induced neuronal survival and tissue repair following SCI. However, PLGA-GDNF NPs intra-spinally injected into the injured spinal cord proximal to the lesion center had no effect on gliosis. The administration of PLGA-GNDF effectively retained neuronal fibers and led to the recovery of hindlimb locomotor performance in rats with SCI, providing a potential strategy for PLGA-GDNF treatment of SCI (Wang et al., 2008). As mentioned above, SCI requires different drugs at different timepoints to inhibit bioactive molecules, which could inhibit neurite outgrowth, and impede the formation of glial scars, facilitating CNS neuron regeneration. The administration of different drugs at different times may result in secondary damage. Therefore, the same team successfully obtained a patent for controlled-release multidrug formulations for SCI (Wang et al., 2009). They designed a core-shell structure of PLGA NPs, in which different drugs can be loaded into the core and shell layers. When the shell disintegrates, the first drug can be continuously released. After the shell has collapsed, the core layer can further disintegrate, thus releasing the second or third drugs. This nanocarrier design can be used for the further treatment of SCI at different times.

## Simple design—Multistep nanomedicine

As well as encapsulating different therapeutics in one vehicle, research has also been carried out to explore the separation of different therapeutics into different individual vehicles. This type of nanosystem is known as multistep NM. Cheng et al*.* developed a nano-theranostic agent (AtkCPTNPs) capable of two-stage size transformation for robust magnetic resonance imaging (MRI)-guided chemoPDT. AtkCPTNPs consists of a pH-responsive polymer, ROS-responsive polycamptothecin-modified iron oxide NPs (IONPs), and aggregation-induced emission PS. In stage one, 90-nm AtkCPTNPs showed excellent stability *in vivo* and accumulated in tumors through the EPR effect. This was followed by pH-induced hydrophobic to hydrophilic transition, which enabled the release of prodrug-modified IONPs, forming large aggregates that were retained in the tumor. Through MRI guidance, PDT was initiated to generate abundant quantities of ROS for therapeutic functions, as well as triggering CPT release for combined chemoPDT. In stage 2, after executing the theranostic functions, large aggregates of IONPs were transformed to a small size for rapid elimination (Cheng G. et al., 2021). Hua et al*.* developed a multistage nanomedicine system for enhanced tumor retention and penetration for bimodal PA/fluorescence imaging and combined radio-phototherapy to inhibit tumor growth and metastasis (Hua et al., 2021). This multistage nanosystem consists of indocyanine green that incorporates chitosan-gold nanoclusters (NCs) (Cs–AuNCs). When administered *in vivo*, the optimal-sized Cs–AuNCs (50 nm) exhibited prolonged circulation and preferentially accumulated in tumor tissues. Owing to their pH responsiveness, large (1,000 nm) aggregates were formed and thus enabled their retention in tumors. Illumination with NIR light not only disintegrated the aggregates into small NPs (5 nm), enhancing their tumor penetration, but also increased blood flow in the tumor. Finally, when irradiated with X-rays, due to the multistage cooperative functions, robust tumor and metastasis inhibition were achieved.

**FIGURE 3:**
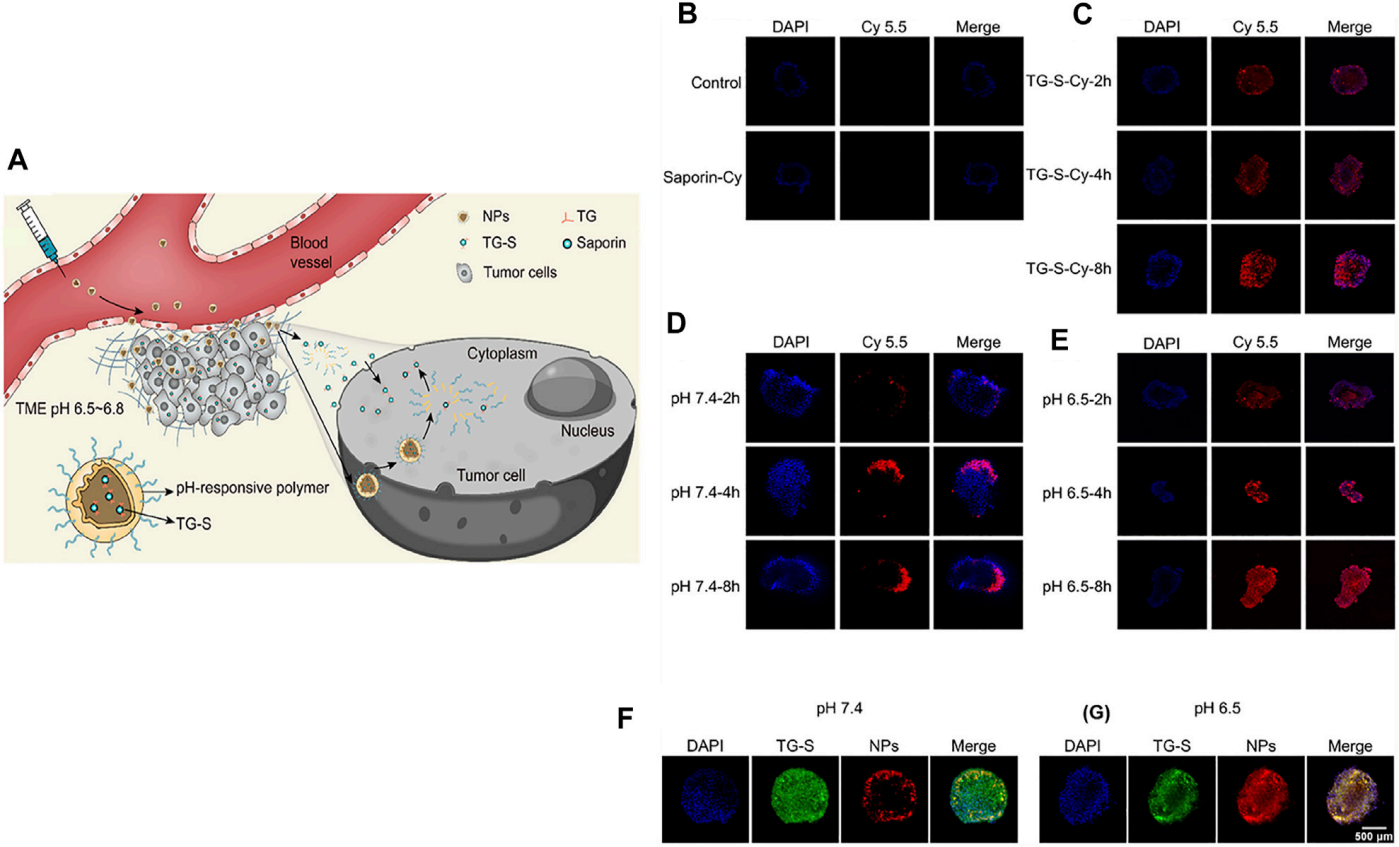
**(A)** Schematic illustration of the formulation of the NPs, release of TG-S in the TME, penetration of tumor tissue, and cellular uptake. **(B–E)** Fluorescent images of A549 tumor spheroids incubated with saporin **(B)**, TG-S **(C)**, and TG-S NPs at pH 7.4 **(D)** or 6.5 **(E)** for 2, 4, or 8 h, as measured by CLSM. The saporin protein was conjugated to Cy5.5 for fluorescence imaging. **(F,G)** Fluorescence images of A549 tumor spheroids incubated with FAM-labeled TG-S (green) and Cy5.5-labeled TG-S NPs (red) at pH 7.4 **(F)** or 6.5 **(G)** for 2 h, as measured by CLSM. Scale bar, 500 μm. Reproduced with permission (Fu et al., 2022). Copyright 2022, American Chemical Society.

Tumor-targeted delivery of carbon monoxide (CO) for tumor killing has been achieved by multistage assembly/disassembly of the nanosystem. The design involves mesoporous silica nanoparticle (MSN)-covered HA loaded with a mitochondria-targeting CO prodrug. This nanosystem was realized by electrostatic assembly (FeCO-TPP@MSN@HA). When administered in an orthotopic tumor-bearing mouse model, the electrostatically assembled nanosystem accumulated in tumors through a combination of EPR and active targeting. A mildly acidic TME promoted disassembly of FeCO-TPP@MSN@HA. The released FeCO-TPP was selectively taken up by mitochondria, followed by ROS-mediated liberation of CO to selectively destroy tumors. Due to the complicated architecture of the lymphatic system and size-restricted reticular structure of lymph nodes, the delivery of therapeutics is both burdensome and challenging (Meng et al., 2020). Schudel et al*.* designed a multistage nanodelivery system that negotiated these obstacles to inhibit lymph node tumors. Toll-like receptor-9-carrying polypropylene sulfide NPs, which include thiol-reactive oxanorbornadiene (OND) linkers in their framework, were developed that demonstrated a high affinity for the lymphatic system. Peripheral administration of the rationally designed nanoplatform was found to be accumulated in draining lymph nodes. In a pH- and solvent-responsive manner, the loaded immune agonist was released and taken up by lymphocyte populations to destroy lymph node tumors (Schudel et al., 2020). Fu et al. (2022) developed a nanoplatform for the cytosolic delivery of proteins for effective tumor accumulation and penetration to inhibit tumor growth. For this purpose, a tetra guanidium (TG) molecule was conjugated to a model protein, saporin, which could facilitate cytosolic delivery (Figure 3). The as-prepared protein conjugate was loaded into a TME pH-responsive polymer that would undergo transition from hydrophobic to hydrophilic, thus liberating the loaded cargo. When administered *in vivo*, the protein-loaded NPs safely delivered saporin in tumors and showed markedly higher accumulation in tumors, enabling their destruction.

## Advances in NCs

Here, we summarize the advances in imaging and therapy applications of NCs. NIR imaging of the gastrointestinal (GI) tract is challenging due to the fluorescence quenching phenomenon. To overcome this limitation, we developed a protein corona containing ribonuclease-A encapsulating gold NCs (AuNCs) that could red-shift the fluorescence emission of NIR-II. The average diameter of the synthesized AuNCs was around 2.2 nm, and they exhibited emission at a wavelength of 1,050 nm with a quantum yield of 1.9%. The *in vivo* imaging potential of AuNCs was tested in both normal and intestinal tumor-bearing mice. Following oral administration, NIR-II fluorescence emanating from the NCs enabled the detection of tumors in the GI tract as small as 2.5 mm, with multi-fold sensitivity (Wang et al., 2020). The inflammatory response associated with PTT is undesirable; to avoid this, Zhou et al. developed ultra-small tungsten NCs (WNCs) via a redox reaction with gallic acid. The as-synthesized WNCs were approximately 2 nm in diameter, biocompatible, and had a photothermal conversion efficiency of 58%. In a mouse tumor-bearing model, intravenously administered WNCs demonstrated excellent therapeutic effects in the presence of 808-nm laser irradiation. In addition, WNCs demonstrated ROS scavenging ability and were able to significantly reduce the formation of cytokines associated with the inflammatory response induced by PTT (Zhou et al., 2020). Li et al. realized the synergistic effects of radiofrequency ablation and transarterial embolization of tumors using core/shell-type NCs. The synthesized nanoplatform consisted of an Au0 core and abundant Au (I) ions as a shell, covered with temperature-responsive poly (N-isopropylamide-co-acrylic acid). In the presence of an RF pulse, the core/shell NCs generated uniform heat that could ablate tumors and at the same time promote temperature-dependent sol-gel transition enabling arterial embolization of tumors. *In vivo*, these synergistic effects were realized in animal models that resulted in the modulation of the TME, which induced favorable immune responses (Lia et al., 2020).

Jia et al. developed an atomically precise gold nanocluster, Au_8_NC (2 nm), that bore levonorgestrel with satisfactory biocompatibility. The synthesized Au_8_NCs, when combined with X-rays, reduced the surviving fraction of cells in a colony formation assay. *In vitro*, Au_8_NCs were sensitized by the ionizing radiation and induced ROS formation under a low dose (4 Gy), which resulted in irreversible apoptosis. *In vivo*, owing to their ultra-small size, preferential accumulation in tumors was achieved when administered intraperitoneally, and they exhibited superior radiotherapeutic effects in the presence of X-rays (Jia et al., 2019). Jia et al. investigated the chirality-dependent radiosensitization effects of AuNCs. For this purpose, L/D AuNCs with a diameter of 2 nm were synthesized, and *in vitro* studies demonstrated that D-AuNCs had superior radiosensitization properties compared with those of L-AuNCs. The authors postulated the following reasons: D-AuNCs exhibited excellent dispersibility, were less toxic, and the formation of ROS was found to be due to the radio-enhancement mechanism. *In vivo*, the nanoclusters demonstrated effective clearance from the body and significant tumor suppression when irradiated with X-rays (Jia et al., 2021). Jiang et al. developed a photothermal agent based on NCs by conjugating indocyanine green (ICG), an FDA-approved fluorescent dye and a photothermal agent, onto glutathione-coated Au_25_. The as-synthesized ICG–GS-Au_25_ was approximately 3.4 nm in diameter and exhibited excellent biocompatibility and photothermal properties. Initially, *in vivo* biodistribution studies showed accumulation of ICG–GS-Au25 in the liver, followed by the dissociation into ICG and Au_25_ in the presence of liver-resident glutathione, with minimal accumulation in healthy tissues. Eventually, Au_25_ was excreted from the body via the renal mechanism, while ICG was metabolized in the liver and excreted in the feces. ICG–GS-Au_25_ predominantly accumulated in the tumor due to the EPR effect and exhibited good photothermal properties in the presence of NIR laser irradiation compared with those of ICG alone (Jiang et al., 2020). Xiao et al. (2020) achieved repair and regeneration in the central nervous system using AuNCs that promoted microglial polarization from an M1 to an M2 phenotype. For this purpose, dihydrolipoic acid-functionalized AuNCs (DHLA-AuNCs) with a diameter of approximately 1.87 nm were prepared. It is generally known that DHLA possesses, and AuNCs can be transported across biological membranes and penetrate the blood–brain barrier. In a microglial cell line, DHLA-AuNCs induced polarization toward the M2 phenotype and suppressed pro-inflammatory pathways. Decreased ROS generation, reduced NF-kB signaling, and increased cell viability was observed. In addition, conditioned medium from DHLA-AuNC-treated glial cells demonstrated neurogenesis in the N2a cell line and an *ex vivo* stroke model.

Sun et al. developed aggregation-induced emission (AIE), PS-embedded gold clustoluminogens for low-dose X-ray-induced PDT. Glutathione-stabilized AuNCs with a diameter of approximately 2.6 nm were assembled with cationic poly (allylamine hydrochloride) to generate AIE-gold clustoluminogens (AIE-AuNCs). AIE-AuNCs were not only sensitized by low doses of X-rays and generated cytotoxic hydroxyl radicals but also produced X-ray-excited luminescence (∼5.2 fold increase compared to Au NCs) to activate PS for PDT. When administered intravenously to tumor-bearing mice, AIE-AuNCs predominantly showed accumulation in tumor tissues through the EPR effect and demonstrated X-ray-induced PDT effects in the presence of low-dose X-rays (Sun et al., 2020). To overcome the depth limitations of conventional PDT, Han et al. developed DHLA-conjugated AuNCs (DHLA-AuNCs) as a TP PDT system (Han et al., 2020). The synthesized DHLA-Au NCs were approximately 1.7 nm in diameter and exhibited superior cross-sectional TP absorption (*σ*_2_∼10^6^ GM) with efficient ROS generation by a type I photochemical mechanism. In HepG2 cells, TP-PDT using DHLA-Au NCs generated copious quantities of ROS, induced lysosomal membrane permeabilization, and brought about mitochondrial morphology change, indicating apoptotic cell death. In tumor-bearing mice *in vivo*, DHLA-Au NCs were intratumorally administered and irradiated with 800-nm laser light. The results showed robust TP-PDT effects with significant inhibition of tumor growth and with no toxic side effects in the mice.

**FIGURE 4:**
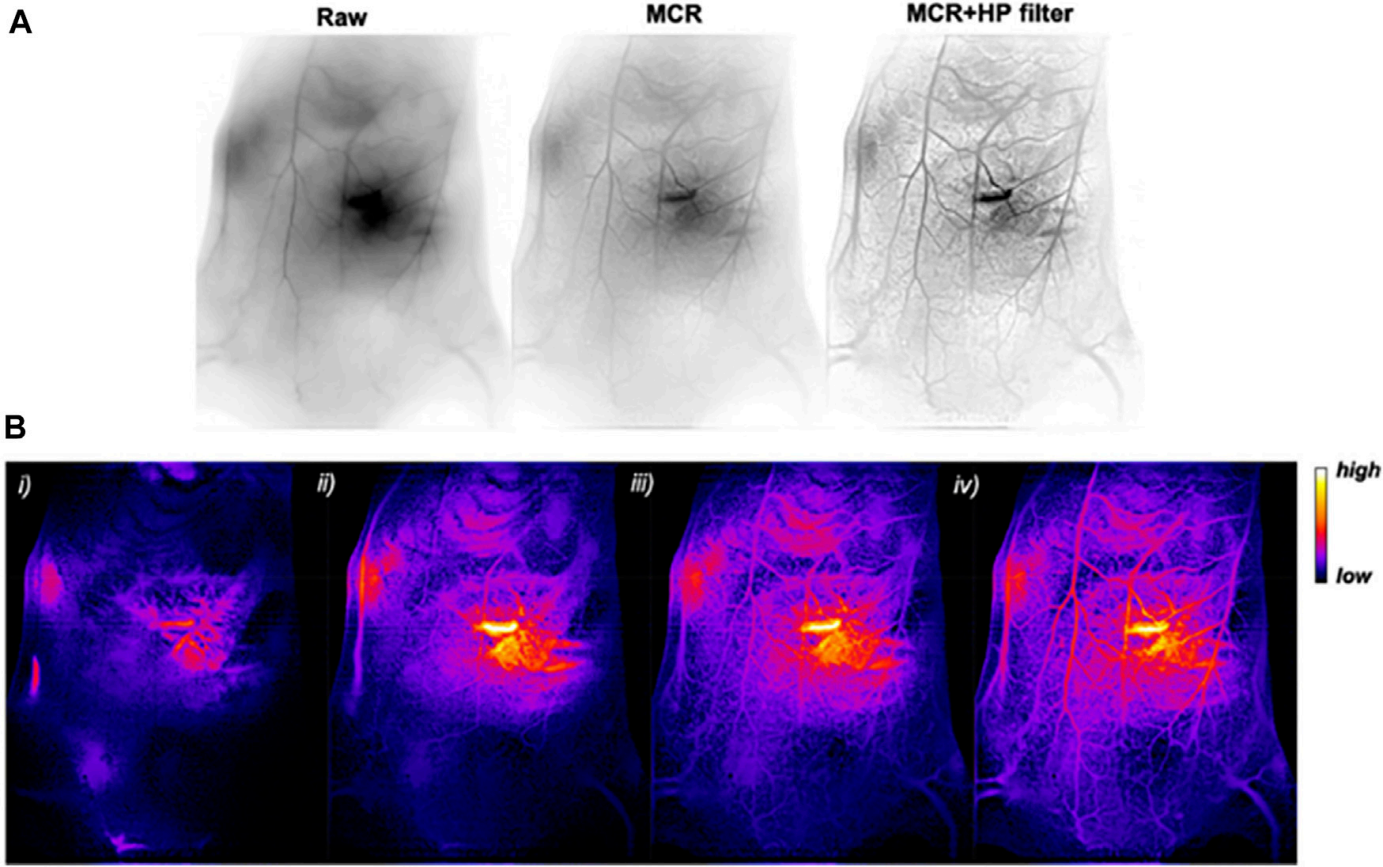
**(A)** *In vivo* SWIR imaging (reverse contrast) of WT 129/Ola mouse vasculature before imaging processing (raw) and after MC restoration (MCR) and an additional filtering (MCR + HP filter). **(B)** MCR + HP filter-treated SWIR images (false colors) (i) 1.5 s, (ii) 5 s, (iii) 25 s, and (iv) 65 s after intravenous injection of an AuMHA/TDT bolus (360 μm; 200 μl). Reproduced with permission (Yu et al., 2020). Copyright 2020, American Chemical Society.

**FIGURE 5:**
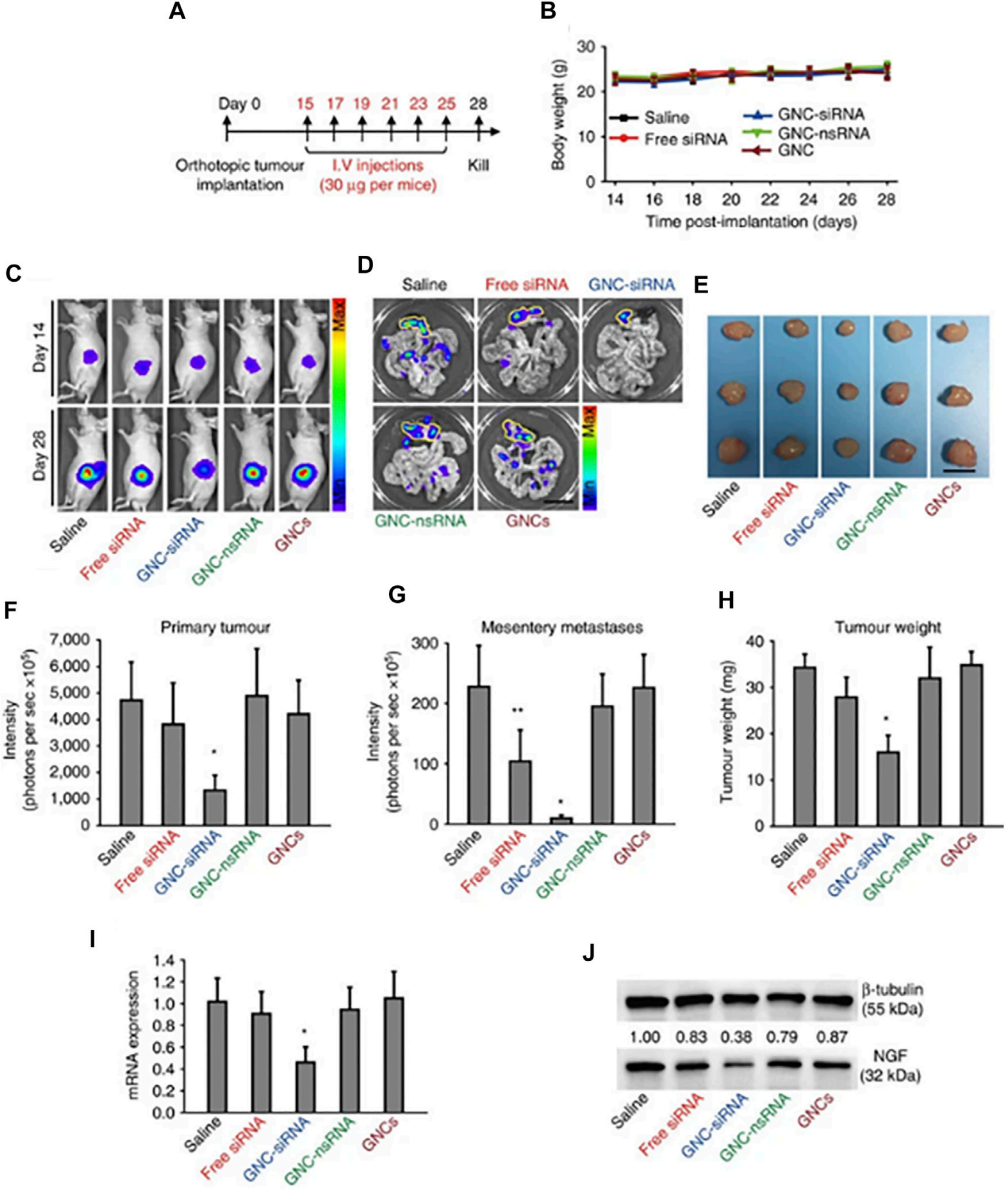
**(A)** Scheme of siRNA treatment. Panc-1-luc cells were injected into the pancreas head of Balb/c nude mice to form orthotopic tumors. After 2 weeks, mice were divided into different groups. Mice received various formulations via tail-veil injections, six times, and were euthanized on day 28. **(B)** Changes in mouse body weight during treatments. **(C)** *In vivo* whole-body bioluminescence images of mice on day 14 and day 28, which indicated the tumor size before and after siRNA treatment. Bioluminescence signals resulted from the interaction of luciferase from Panc-1-luc cells with D-luciferin injected into the mice before imaging. **(D)** *Ex vivo* bioluminescence images of orthotopic pancreatic tumors and tumor metastases into mesenteries on day 28. Yellow lines indicate the locations of primary tumors in the pancreas. Scale bar, 1 cm. **(E)** Tumor images on day 28. Scale bar, 5 mm. **(F)** Quantification of *in vivo* bioluminescence to evaluate the primary tumors in mice on day 28. **(G)** Quantification of tumor metastases by the sum of *ex vivo* bioluminescence detected in the mesenteries on day 28. **(H)** Weight of the isolated tumors. **(I)** *NGF* mRNA and **(J)** NGF protein expression levels in orthotopic tumors. Reproduced with permission (Lei et al., 2017). Copyright 2017, Springer Nature.

Recently, Yu et al. developed atomically precise AuNCs with an anisotropic surface coating using a short dithiol PEG (Figure 4). When administered *in vivo*, AuNCs with a quantum yield of approximately 6% in the shortwave infrared (SWIR) spectrum enabled whole-body vascular imaging, with enhanced resolution assisted by a battery of Monte Carlo (MC) image processing (Yu et al., 2020). Lei et al. (2017) developed an AuNC-small interfering (si)RNA complex to silence the gene expression of nerve growth factor (NGF) and destroy pancreatic tumors. To demonstrate this idea, glutathione and oligoarginine stabilized AuNCs were synthesized and loaded with siRNA (encoded for NGF) through an ionic complex. *In vivo*, the AuNC-siRNA complex showed increased stability, prolonged circulation, and enhanced accumulation in tumors. The efficacy of this siRNA formulation was tested in subcutaneous, orthotopic, and patient-derived xenograft pancreatic tumor models (Figure 5). The results showed effective tumor growth inhibition in all tested tumor models, due to the knockdown of NGF gene expression by the siRNA formulation and with no apparent toxicity. Liu et al. developed atomically precise AuNCs (approximately 1.7 nm in diameter), comprising 25 gold atoms and 18 peptide ligands, for NIR II imaging. To test the NIR II imaging performance *in vivo*, the AuNCs were intravenously administered in a mouse stroke model (Liu H. et al., 2019). The results showed that the intensity of neovascularization in the damaged left brain increased with time and that more arterial vessels were apparent in the left brain compared with in the right brain. Additionally, metastasis imaging potential was investigated following intravenous administration of AuNCs in a 4T1 tumor metastasis mouse model. The primary tumor and metastasis could be clearly observed 5 min post-intravenous injection. Ultimately, after 48 h the AuNCs were excreted from the body via the renal filtration mechanism with an efficiency of approximately 86% and without any toxic effects. Colombé et al. (2019) synthesized and utilized AuNCs for fluorescence image-guided surgery of head and neck tumors. To investigate its potential, AuNCs were coated with either zwitterionic or PEG ligands. For the *in vivo* biodistribution study, ligand-coated AuNCs were systemically administered to tumor-bearing mice and their accumulation in organs was observed after 5 h. The results showed clear optical signals in the liver, spleen, kidney, and, most importantly, the skin and tumors. The authors found that PEG-coated AuNCs were rapidly eliminated via the renal mechanism. Optical image-guided surgery in orthotopic tumor-bearing mice following the administration of ligand-coated AuNCs allowed the detection and removal of otherwise undetected tumor residues.

Sultan et al. synthesized PET-responsive AuNCs (Cu64-AuNCs) and investigated their delivery across the blood–brain barrier in the presence of focused ultrasound (FUS). Cu64-AuNCs with positive, negative, and neutral charges were synthesized and their *in vivo* PK were evaluated. Among the three charges, neutral Cu64-AuNCs exhibited optimal organ accumulation properties. Following FUS irradiation in a wild-type mouse, PET imaging of Cu64-AuNCs showed their penetration, retention, and diffusion in the brain. Further analysis showed intra-brain distribution patterns of all the Cu64-AuNCs, and histopathology studies showed no observable toxicity (Sultan et al., 2018). For the targeted RT of prostate cancer, Luo et al. (2019) developed Au25-NCs, which were labeled with prostate-specific membrane antigen targeting ligand, CY-PSMA-1. Targeted Au25-NCs demonstrated a high degree of specificity for prostate cancer cells and exhibited radiotherapeutic performance under X-ray irradiation. The *in vivo* biodistribution in prostate tumor-bearing mice showed significant accumulation of targeted Au25-NCs in the prostate tumors at 4 and 24 h. In the presence of X-rays *in vivo*, targeted Au25-NCs demonstrated robust radiotherapeutic effects with a significant reduction in tumor volume while retaining the body weight of mice that underwent treatment, with no toxicity. Song et al. (2021) developed NIR II AuNCs for *in vivo* tumor-targeted imaging and the efficient labeling of proteins. For this purpose, sub-2-nm AuNCs were prepared and stabilized with biocompatible cyclodextrins (AuNC-CD). Through hydrophobic interactions, CD can form complexes with amino acids in protein/antibody structures without affecting their structural integrity. AuNC-CD enabled the efficient labeling of proteins, which improved their stability and prolonged their circulation *in vivo*. When they were intravenously administered, NIR II imaging showed an increased accumulation of AuNC-CD in the kidneys from 10 to 90 min and in the bladder from 10 min to 3 h. Moreover, after 24 h, fluorescence signals were not observed in the bladder, which indicates there was efficient renal clearance. Finally, using NIR II imaging in a tumor-bearing mouse model, intravenously administered, targeted AuNC-CD predominantly showed accumulation in tumors over time. Inspired by the famous Folin–Ciocalteu assay, Zhou et al. developed biocompatible and renal clearable monodispersed, tungsten-based polyoxometalate NCs (W-POM-NCs) via a redox reaction in an alkaline solution (Zhou et al., 2020). The synthesized W-POM-NCs were approximately 2 nm in diameter and showed a photothermal conversion efficiency of 58%, as well as non-inflammatory properties. In tumor-bearing mice *in vivo*, intravenously administered W-POM-NCs showed specific accumulation in tumors, resulting in excellent photothermal performance and reduction in inflammation related to the PTT by scavenging the ROS. Due to their ultra-small size, the W-POM-NCs were efficiently excreted from the body via renal mechanisms.

## Dendrimer nanoparticles

The induction of systemic toxicity is one of the major limitations of cancer vaccines. In an attempt to resolve this, amphiphilic dendrimer NPs were developed encapsulating Ce6 as a light-activable immune adjuvant (LIA) (Wang Y. et al., 2021). When irradiated with NIR light, tumor cell lysis occurred, followed by the release of tumor-associated antigens; hypoxia was also induced. This triggered the structural transformation of the dendrimer NPs, via which 2-nitroimidazole was converted into 2-aminoimidazole, which has the ability to activate dendritic cells. *In vivo*, LIA inhibited primary tumors and induced a robust immune memory effect to prevent metastasis. Therapeutics face a difficult task to reach pancreatic tumors due to their dense stroma. Wang et al. developed a glutathione-covered *cis*-platin (CPT)-dendrimer conjugate through an ROS-responsive bridge (Wang et al., 2021). The glutathione in the dendrimer can be converted to primary amine by glutamyl transpeptidase (GGT), which is highly expressed in pancreatic tumor endothelial cells. *In vivo*, the dendrimer in the presence of GGT enabled their surface with positive charge. As a result, dendrimer achieved deep tumor penetration through the mechanism of caveole-mediated endocytosis and transcytosis. Inside the tumor cells, CPT was released by ROS, inducing apoptosis and destroying pancreatic tumor cells. Zhang et al. developed a cluster-bomb like NP (CPIM) that integrates two dendrimers loaded with different drugs (a PTT/PDT agent and idoleamine 2,3 dioxygenase (IDO) inhibitor) bridged by an ROS-responsive linker covered with chondroitin sulfate (CS). In a tumor-bearing mouse model, CPIM accumulated in tumors by a passive targeting mechanism, and CS was digested by hyaluronidase. In the presence of laser light, PTT/PDT actions were established, generating ROS that disintegrated CPIM to release the drug-loaded dendrimers. Through both passive diffusion and an active transport mechanism, the dendrimers achieved deep tumor penetration. The therapeutic actions induced an immune response via immunogenic cell death that inhibited the growth of primary, bilateral tumors and metastasis (Zhang et al., 2021).

## Mesoporous silica nanoparticles

Cheng et al. developed high intensity focused ultrasound (HIFU) and magnetic resonance imaging (MRI) responsive mesoporous silica nanoparticles (MSN) covered with bi-functional poly(ethylene glycol). A commercially available gadolinium (Gd)-based MRI contrast agent was loaded inside MRgHIFU as an imageable component. Under HIFU, poly (ethylene glycol) (PEG) was cleaved, and the cargo release was monitored by MRI with no substantial increase in temperature observed. The three-dimensional (3D) capabilities of HIFU and MRI enable the cargo release to be highly localized at the focal point of the HIFU. The cargo release was also monitored with *ex vivo* MRI, which showed a positive correlation between cargo release and the T1 signal (Deng et al., 2021). Wang et al. developed a multifunctional theranostic core/shell NP that performed as an immunomodulatory nanozyme. The core consists of iron carbide NPs covered with copper ions, and together they serve as a PTT, chemodynamic, and MRI agent. The shell is a mesoporous silica coating loaded with immune adjuvant that can be released in a pH-/temperature-dependent fashion, while the outer surface is covered with a fluorescent imaging agent and a tumor targeting moiety. *In vivo*, when irradiated with laser and through the combined action of PTT and CDT, tumors were destroyed and released antigens to initiate an immune response. Furthermore, the release of the immune adjuvant in a stimuli-responsive fashion compounded the immune therapy effects and provided a robust systemic immune response (Wang et al., 2022).

## Peptide-based nanostructures

Lopez-Silva et al. explored the feasibility of using a self-assembling multidomain peptide (MDP) hydrogel for nervous system regeneration. For this purpose, different types of MDPs that contained various extracellular matrix components and growth factors that self-assembled into nanofibrous, injectable hydrogels were developed. An *in vitro* primary neuronal culture performed on MDP-coated cover slips showed that a lysine-containing MDP promoted neurite growth. In a rat model with sciatic nerve injury induced to study the MDP’s bioactivity, the injected MDP degraded over time and promoted nerve regeneration and remyelination (Lopez-Silva et al., 2021). Chakraborty et al. developed a cell-adhesive RGD-based self-healing hydrogel containing two F-moc groups. The as-prepared hydrogel showed excellent mechanical stability owing to the *π*-stacking interactions of the two F-moc groups. Molecular dynamics analysis revealed that the two F-moc groups and lysine formed a hydrophobic core, while RGD was present on the surface. To further impart mechanical stability and conductivity, poly aniline was integrated into the hydrogel. The composite hydrogel showed antibacterial and DNA-binding properties and, importantly, promoted the assembly of cardiomyocytes into contracting cells. The design and development of biomaterials that promote tissue regeneration following an injury is highly desired (Chakraborty et al., 2021). Alcarez et al. developed supramolecular, amphiphilic peptide fibrils that included two peptide sequences, for nervous system regeneration. Two biological sequences, one to promote cell proliferation and survival and the other to mitigate glial scarring, were placed on the terminus of two different alkyl peptides. The authors showed that by slightly mutating the non-bioactive domains in the monomers, intensified motions of the molecules within the scaffold fibrils could be achieved. As a consequence, functional recovery of the nervous system was observed in mice with severe SCI (Tang et al., 2019).

## The pharmaceutical industry’s view of the translational challenges of nanomedicine

The first liposomal nanomedicine, Doxil™, was approved in 1995, and since then drug nanoformulation has been continuously developed in clinical trials. Perhaps the most prominent to date are lipid-based NPs, such as liposomes and polymers (Ventola, 2017). The aims when developing drug nanoformulations are to reduce side effects and achieve sustained release for effective treatment to avoid the need for multiple doses. In the treatment of ovarian cancer, sustained-release Dox nanoformulations, such as Doxil™ and LipoDox replaced the traditional Dox, which requires a single shot each day for effective treatment, and reduced the dosage of Dox required. One study found that increasing the drug content in the polymer conjugate may retard drug release and further stabilize NPs, providing enhanced protection for the drug and thus improving both its PK and efficacy (Ernsting et al., 2011). Therefore, the drug content of nanocarriers is a critical factor to achieve sustained release and avoid multiple doses. The physiochemical properties of active pharmaceutical ingredients (APIs) and the characteristics of nanocarriers may affect the drug content of nanocarriers. As a consequence, establishing the interactions between the physiochemical properties of the API and the chemical composition of the nanocarrier, as well as the synthesis methods, greatly impacts the drug release rate.

## Biodistribution, stability, and biocompatibility of nanoparticles

The study of measuring the concentration field of therapeutics in the major organs, administered over a period of time, is referred to as pharmacokinetics (PK). To assess the concentration of injected therapeutics, longer time periods are usually favored. The data obtained from PK studies can be used not only to learn about the behavior of pharmaceuticals but also to determine the dose required for optimal blood concentration without any undesirable side effects. The efficacy and toxicity of therapeutic agents are largely based on their serum concentration levels. Greater accumulation of therapeutic agents in the targeted or intended organs leads to better therapeutic effect, whereas large quantities of therapeutic agents distributed in unintended organs leads to toxicity. Water-soluble therapeutics when administered intravenously undergo rapid elimination from the body via renal filtration. Hydrophobic therapeutics are metabolized in the liver before being eliminated from the body. Therapeutics that are loaded inside NPs are protected from being metabolized in the liver, while the binding of serum proteins (opsonins) can increase their size and prevent their renal elimination (the cut-off size is 5.5 nm). The pore size of the endothelial wall determines the entry of NPs into tissues; for example, disorganized, leaky endothelial walls allow enhanced accumulation of NPs in tumors through a phenomenon called the enhanced permeability and retention (EPR) effect. Major organs, such as the liver, spleen, and bone marrow, have also been shown to take up large quantities of NPs. This is due to the macrophages present in these tissues that form part of what is known as the reticuloendothelial system or the mononuclear phagocyte system and which is responsible for eliminating foreign particulates and macromolecules (Opanasopit et al., 2002; Brannon-Peppas and Blanchette, 2004; Choi et al., 2007). Souris et al. observed the biodistribution and excretion profiles of positively and negatively charged MSNs. Indocyanine green-labeled MSNs of identical sizes were intravenously administered to male nude mice or Sprague–Dawley rats and monitored using *in vivo* fluorescent imaging. The results showed that initially, both positively and negatively charged MSNs were taken up by the liver. However, the former were rapidly mobilized into the GI tract, and the latter were found to be residing in the liver. In addition, inductively coupled mass spectrometric analysis of harvested organs revealed that the biodegradation of MSNs into orthosilicic acid was initiated 3 days post-administration. The authors postulated that positively charged MSNs were electrostatically bound by serum proteins and hence amenable to hepatobiliary excretion into the GI tract (Souris et al., 2010).

Dogra et al. studied the effect of MSN size, surface charge, and route of administration on their biodistribution and clearance kinetics using *in vivo* single-photon emission computed tomography. For this purpose, MSNs with an average size of 50 nm were modified with cationic groups (poly(ethyleneimine) (PEI)) and quaternary amines (QA)) and trimethyl silane (TMS) (neutral). In addition, MSN-TMS was evaluated for nominal sizes of 25, 90, and 150 nm. PK analysis showed that irrespective of the route of administration (intravenous or intraperitoneal.), MSNs of smaller size showed greater bioavailability, while the peritoneal absorption of MSNs following intraperitoneal injection was independent of size. Cationic MSN-PEI showed poor circulation *in vivo* compared with a QA counterpart, probably due to their rapid uptake by the liver and spleen. Interestingly, MSN-QA showed a better total excretion profile compared with MSN-PEI and MSN-TMS. A widely used method to avoid MPS uptake is to cover the surface of the NPs with PEG. PEG is inert, hydrophilic, and is known to significantly reduce opsonization and improve PK (Dogra et al., 2018). For instance, He et al. used *in vivo* fluorescence imaging to study the biodistribution and excretion profiles of surface-modified silica NPs. For this study, fluorescent SiNPs with different functional groups (Si-OH, Si-COOH, and Si-PEG) and an average size of 45 nm were synthesized. Following intravenous administration in mice, the biodistribution of the as-synthesized SiNPs was observed using *in vivo* fluorescence imaging. The results showed that, irrespective of the functional group, all SiNPs were eliminated from the blood circulation. However, distinct half-lives and distribution in major organs were observed for different functional SiNPs. Si-PEG NPs showed a substantially prolonged circulation half-life (t1/2 = 180 ± 40 min) compared with Si-OH NPs (t1/2 = 80 ± 30 min) and Si-COOH NPs (t1/2 = 35 ± 10 min). In addition, *in vivo* fluorescence imaging showed that all three SiNPs were partially excreted via the renal filtration mechanism (He et al., 2008).

## Effect of administration route on absorption, distribution, metabolism, and excretion

Several studies that investigated administration routes showed the administration route could improve the biodistribution, which may alter the *in vivo* fate of NPs (Battaglia et al., 2018; Chenthamara et al., 2019). For instance, Dölen et al. investigated the effect of nanovaccine (NV) administration route on the induction of an immune response. The NV was made of poly (lactic-co-glycolic) acid loaded with antigen and invariant natural killer T (iNKT) cell agonists (Dölen et al., 2020). Intravenously administered NV accumulated in the liver and spleen, indicating a robust immune response, whereas intranodal or subcutaneously administered NV could barely reach the lymphoid tissues. In addition, the authors found that the intravenous route was safe, and doses of up to 50 mg/kg did not cause any toxicity; they also discovered that the NV synergized with immune checkpoint modulation for better tumor control. Fu et al. systematically investigated the effect of various administration routes on the *in vivo* fate of 110-nm SiNPs. The results revealed that, following oral administration, SiNPs were absorbed by the intestine into the portal vein and transported to the liver, with this route having a higher absorption rate compared with those of intramuscular or hypodermic administration. Intravenously administered SiNPs accumulated in the lung, intestine, and muscle after 24 h (Fu et al., 2013). The SiNPs were subsequently taken up by the liver and spleen. Irrespective of the administration route, SiNPs were excreted in the urine and feces, and histopathological examination of the vital organs showed no toxic effects. Bednarski et al. (2015) used 25-nm AuNPs and studied their tissue distribution pattern following intravenous and oral administration. The results showed that most of the intravenously administered AuNPs accumulated in the liver by phagocytosis. As a result, a very small quantity of gold was observed in the feces and urine over a 10-day period. On the other hand, orally administered AuNPs showed the least accumulation in the organs, and most were eliminated in the feces 4 days post-administration.

## Lipid NPs and COVID-19 vaccines

Recently, FDA authorized the Moderna and Pfizer-BioNTech COVID-19 vaccines for children down to 6 months of age. Both of these vaccines, which were developed at an unprecedentedly rapid pace, consist of messenger RNA (mRNA) encapsulated within lipid nanoparticles (LNs). Due to their large size and strong negative charge that electrostatically repulses cell membranes, mRNA molecules are intrinsically unable to cross biological membranes, thus preventing uptake. Therefore, a delivery system is needed for the safe delivery of mRNA into target cells, which will then allow the mRNA to be transcribed into proteins, producing copious quantities of neutralizing antibodies. LNs have been well studied as a carrier for nucleic acids, with their physiochemical properties such as surface charge, composition, size, loading efficacy, cell uptake, release behavior, dosage, and route of administration all thoroughly investigated. The vast experience and knowledge gained from this research accelerated the development of optimal LNs for the delivery of mRNA. LNs are spherical and comprise helper lipid, cholesterol, and PEG, with pH-dependent charge transition properties. Under low pH, the surface of LNs is positively charged by ionizable lipids but remains neutral at physiological pH. These properties enable LNs to ensure safe passage for mRNA through endosomes with subsequent release into the cytoplasm. The ratio of individual components of LNs will largely determine their *in vivo* efficacy and safety.

## Regulatory challenges

The ineffective treatment of disease with single functional drug carriers has promoted the development of multifunctional nanomedicines (NMs), such as theranostics and multitherapy NPs. However, multifunctional NM in clinical settings has rarely been studied. Theranostic NMs provide diagnostic and therapeutic functions in a single nano-entity for precision medicine and provide potential applications for numerous types of research. In 2018, ^89^Zr-Df-CriPec® docetaxel for PET imaging of solid tumors was tested in Phase 1 clinical trials. This nanomedicine consists of polymeric NP that contains imaging contrast agent and a drug, investigated in clinical trials Therefore, theranostic NPs represent a promising nanocarrier for clinical development. Multitherapy NPs that encapsulate two or more drugs in a single nano-object are another type of multifunctional nanomedicine. In 2017, FDA granted accelerated approval for the combination of daunorubicin and cytarabine liposomes (Vyxeos®) to treat high-risk acute myeloid leukemia (AML). This multitherapy NP is the first approved treatment specifically for patients with certain types of high-risk AML. Interestingly, both theranostic and multitherapy NPs in clinical development are advancing slowly despite rapid developments in academic research.

The process of approval of a new chemical entity (NCE) can be broadly divided into the pre-clinical and clinical phases. The purpose of clinical trials is to confirm whether an NCE is effective and nontoxic in the human body. Preclinical studies determine whether an NCE has the potential to cause serious toxicity, prior to any clinical trials being conducted. Pharmacological toxicity and safety tests in animals can ensure humans are not exposed to any toxicity due to the drug substance. Chemistry, manufacturing, and control (CMC) is a crucial element of preclinical drug development. It includes the establishment of physiochemical properties, stability testing, and analytical methods, as well as dosage form design and optimizing the manufacturing process for scale-up. NMs include various formulations of drug nanocarriers, such as nanomaterial-encapsulated drugs and nanomaterials that possess therapeutic, diagnostic, or theranostic capabilities. Overall, the more complicated the structure of an NM, the more difficult it is to achieve CMC. For instance, the physiochemical properties of NMs can be difficult to control during scale-up or because of variations in batch-to-batch synthesis. Therefore, the manufacturing of NM at an industrial level requires precise control of physiochemical properties during large-scale synthesis. In addition to the physiochemical properties of scaled-up NM, the stability of the nanocarriers should take into account any interactions between the API and the nanomaterials or interactions between the nanocarriers. In 2017, FDA released draft guidance for industry, entitled “Drug products, including biological products, that contain nanomaterials.” This document provides guidance on the development of drug products for use in humans and specifies/suggests several items that should be taken into account when using nanomaterials in CMC specifications. Specifically, it is necessary to provide information about the nanomaterials, determine the physiochemical properties that may affect the properties of the product, and offer an appropriate analytical method to determine the physiochemical properties. Drug products that contain nanomaterials must be manufactured in accordance with current good manufacturing practice (cGMP) and should have dissolution/*in vitro* release methods capable of discriminating formulation and manufacturing differences that may affect the clinical performance of the drug product. If the drug product must be diluted prior to use, the dilution medium may affect the colloidal stability of the nanomaterial and trigger the release of the active ingredient. In-use stability studies at clinically relevant concentrations and under relevant storage conditions may also be required. However, some of the previously mentioned NMs are not covered by this draft guidance because it does not apply to biological products composed of proteins, cells, viruses, nucleic acids, or other biological materials that occur naturally at particle sizes ranging up to 1,000 nm. This means that the current guidance does not comprehensively include all formulations of NM, thus they need to be discussed on a case-by-case basis.

Due to the lack of regulatory experience with nanomedicine, both drug developers and regulatory authorities, such as FDA and the European Medicines Agency (EMA), must allocate extra efforts toward developing or regulating drug nanoformulations, thus making drug nanoformulation more challenging than investigational new drug (IND). The International Organization for Standardization (ISO) and ASTM International have developed and published several documents specifically for the standardized physiochemical and biological characterization of nanomaterials. For instance, ISO published a technical report about toxicological tests on nano-objects in 2012. This technical report provided several physiochemical properties that were considered critical for assessment prior to toxicological tests and identified which parameters should be measured to assess the physiochemical properties of nanomaterials. For example, particle size measurement should always be performed under the same conditions, while the determination of particle size distribution is important for assessing the effect of particle size on toxicology. Halamoda-Kenzaoui et al*.* compiled the information requirements released by regulatory scientists thus far and mapped them against available standards that could be relevant for NM (Halamoda-Kenzaoui et al., 2019).

To improve the clinical outcome of NM drugs, one promising approach is to strive toward the development of personalized medicine. However, pharmaceutical companies are reluctant to invest in NM drugs due to the various challenges associated with them. The design of NMs is complex due to their composition and multiple components, meaning multi-step synthesis processes are necessary. These require extended periods of time to complete, which significantly increases the production costs. Following production, there are no standard methods for the purification of NMs. Laboratory techniques such as evaporation and ultrafiltration cannot be implemented in industry due to residual solvent contamination and, depending on the NM design, distinct purification methods must be adopted. At large scales this is challenging, raising questions about economic feasibility. Moreover, bulk production might alter the physiochemical properties of an NM, such as its size, shape, stability, crystallinity, or surface area, which could profoundly influence its biodistribution and metabolism. An even greater threat is the loss of biological activity for NM drugs involving antibodies, genes, nucleic acids, etc. In the traditional way, NM parameters must be fine-tuned to either label or encapsulate the API. In the future, to reduce the cost of production, APIs could be produced with one or more functional groups for facile conjugation with nanomaterials, which may reduce the design complexity and the number of steps involved in scale-up. One of the important parameters to be considered in NM drugs is their cost-effectiveness compared with their generic counterparts (that are clinically available). It is always difficult to balance high production costs with the number of patients willing to buy a product, which may eventually force companies to discontinue their product. The intense preclinical research into NM drugs, their high cost of production, and the tentativeness of pharmaceutical companies to invest due to uncertainty in clinical translation has led to the elevated market price of NM drugs.

## Toxicity of nanoparticles

Traditionally, the toxicity of small molecule drugs is evaluated based on their administration route, concentration, and dose time and frequency. However, to comprehensively evaluate the toxicity of NPs their physiochemical properties, such as size, shape, material, composition, surface charge, etc., must be included. This is because any alterations to their physiochemical properties could drastically affect their *in vivo* biodistribution, clearance, and toxicity. Toxicity evaluations of NPs are crucial for their clinical translation, with cell culture and animal models routinely used for their assessment. Cell culture methods use animal and human cell lines for toxicity evaluations such as cytotoxicity assays, proliferation assays, oxidative stress assays, etc., and are useful because of their scalability, low cost, speed, convenience, and minimal ethical issues. However, due to their inability to mimic *in vivo* conditions, results from cell culture studies cannot be used to predict toxicity outcomes in animals or humans (Muhr et al., 2014; Elci et al., 2016; Poon et al., 2019). Several animal models have been used to evaluate the *in vivo* toxicity of NPs, involving various methods, including biodistribution, clearance, hematology, serum chemistry, and histopathology. Nonetheless, there are still limitations associated with predicting adverse effects and toxicity responses. An interesting approach is the use of computational nanotoxicity, which could provide a link between cell culture, animal models, and humans. The advantage of such studies is that they are rapid, have the potential to screen larger numbers of NPs, and, importantly, they could reduce the need for experimental testing and are thus potentially economical. Despite this, several challenges that require attention remain, such as the absence of standard protocols for nanotoxicity testing and vast variations in published data with respect to physiochemical characterization, administration routes, differing data quality, etc. We summarized the examples of NP related toxicity in humans (Oh et al., 2016; Furxhi et al., 2019; Kolanjiyil et al., 2019).

Khan et al. investigated hypersensitivity reactions (HSR) associated with the administration of Doxil (a PEGylated liposomal doxorubicin formulation) in 29 human patients. The results showed that second to third grade HSRs were observed in 45% of the patients with formation of antibody (SC5b-9) within 10 min following infusion. The study also found a direct correlation between complement activation and HSRs (Chanan-Khan et al., 2003). The pathology of Alzheimer’s disease (AD) is closely associated with the accumulation of high concentrations of magnetite (Fe_3_O_4_) iron. Pankhurst et al. analyzed brain tissue samples obtained from 11 patients with AD for their magnetic properties. Magnetometry studies revealed that through biomineralization sub-20-nm Fe_3_O_4_ NPs were formed within the 8-nm iron-rich protein ferritin. The authors speculated that the formation of Fe_3_O_4_ NPs may be linked with aging and that it becomes abnormal in AD (Pankhurst et al., 2008).

In another study, Mills et al. evaluated the effect of combustion-derived NPs in promoting cardiovascular ailments. For this study, 16 healthy volunteers were exposed to diesel exhaust; blood flow and biomarker analysis showed increased systolic blood pressure and attenuated vasodilation as a result of vascular oxidative stress (Mills et al., 2011). Khatri et al. studied the toxic effects on nine healthy individuals of NPs emitted by photocopiers. For this study, the subjects were exposed to NPs (30,000 NPs/cm^3^) from photocopiers for 6 h per day for a total of 3 days. Nasal lavage and urine samples were collected at various time points and analyzed for 14 cytokines and 8-hydroxydeoxyguanosine (8-HDG). From the results, 2- to 10-fold increases in the 8-HDG and pro-inflammatory cytokine levels were observed due to upper airway inflammation and oxidative stress. Aktetpe et al. tested the toxic effects in 76 silver jewelry workers of chronic exposure to silver. Blood samples were obtained from the participants, and several parameters, such as serum total antioxidative status (TAS), total oxidative status (TOS), total thiol content, and ceruloplasmin levels compared with non-exposed human participants, were evaluated. The results showed an accumulation of severe DNA damage and high serum levels of TOS and oxidative stress index (Aktepe et al., 2015).

## International organization for standardization test methods and guidance for nanomaterial physiochemical characterization and toxicity

Medicinal products must be characterized for their safety and efficacy before being entered into clinical trials. These studies involve both *in vitro* and *in vivo* tests that help to determine the optimal dose and reduce any risks. Guidelines for the preclinical safety assessment of small molecule drugs were released by the International Conference on Harmonisation of Technical Requirements for Pharmaceuticals for Human Use (2009). However, these guidelines cannot be applied to nanomedicines due to their intrinsic properties, such as a highly reactive surface due to their large surface area, charge, adsorption ability, and increased tendency to react with biomolecules such as proteins. Several testing methods for the evaluation of toxicity of NPs have been released by ISO. For instance, 5-(and 6)-chloromethyl-20,70-dichloro-dihydrofluorescein diacetate assays for the assessment of NP-induced intracellular ROS production in RAW 264.7 macrophage cells (ISO/TS 19006:2016) and electron spin resonance as a testing method for ROS formation (ISO/TS 18827:2017). Endotoxins are an important type of biological impurity that affect preclinical grade NPs and which must be completely excluded because they may affect toxicity and efficacy results. An *in vitro* Limulus amoebocyte lysate test method was published to detect endotoxins in nanomaterial samples (EN ISO 29701:2010).

One of the crucial features of NPs is their ability to load drugs and release the free drug in target tissues. The drug-loading efficacy and drug-releasing profile of NPs must be evaluated under both *in vitro* and *in vivo* conditions to determine the safety and efficacy compared with the free drug. An ideal NP-based drug delivery system must be able to load a high concentration of drugs, retain them during *in vivo* circulation, and release them in the intended tissue. Unfortunately, until recently there have been no standard methods available to determine loading efficacy and drug release from NPs. Skoczen et al. developed a reliable and promising method to measure drug release from NPs. For this purpose, an active drug labeled with a stable isotope was prepared and spiked into plasma containing non-labeled active drug encapsulated by NPs (Skoczen et al., 2015). Isotope-labeled drug is in equilibrium with protein similar to drug released from NP. Ultrafiltration of the plasma samples separates the free drug, and mass spectrometry analysis of the ultrafiltrate allows the accurate measurement of loaded and non-loaded drug fractions.

ISO has developed and released standardized test methods for the physiochemical and biological characterization of NPs. International standards (IS), technical reports (TR), and technical specifications (TS) are the three levels of ISO documents. An IS, the main product of ISO, requires approval from national standardization authorities. A TR is an informative document, such as references and explanations, while TS comprise an intermediate document published before the development of IS. Physiochemical characterizations such as size, shape, surface charge, composition, hydrophobicity, and stability, must be tested for NP-based medicines as a part of a preclinical evaluation. These characterizations must be performed in relevant biological media to represent critical factors such as aggregation, sedimentation, dissolution, and opsonization. ISO have released several guides and test methods for the physiochemical characterization of NPs, including dynamic light scattering (ISO 22412), laser diffraction methods (ISO 13320-1) and ISO 13322 (Image analysis) (ISO/TC 24, 1999, ISO/TC 24, 2014), surface characterization of gold NPs by Fourier transform–infrared spectroscopy (ISO/TS 14101:2012), and zeta potential measurements of colloidal systems (ISO 13099-1:2012, -2:2012, -3:2014).

## FDA guidance on liposome (LPM) drug products

FDA released final guidance for the industry on LPM drug products (FDA-2016-D-2817: 2018), which explains the important information that should be submitted with any new drug application (NDA) or abbreviated new drug application (ANDA) to the Center for Drug Evaluation and Research (CDER). LPMs are structures with a lipid bilayer formed by molecules that have both hydrophilic and hydrophobic compartments. LPMs are typically highly biocompatible and are therefore used to improve the aqueous solubility of hydrophobic drugs, reduce their systemic toxicity, and alter the pharmacokinetic properties of a drug.

## Chemistry, manufacturing, and controls

The FDA guidance outlines that NDAs and ANDAs should list all of the drug components by their established names, along with the quantity of each drug component and the molar ratio of lipid components. The physiochemical properties of LPMs determine their quality and efficacy, so extensive characterizations, CQA, are required of their features, such as morphology, net charge, viscosity, size, phase transition temperature, *in vitro* drug release from LPMs using standard protocols, and the leakage rate of the drug from LPMs throughout their shelf life. In addition, manufacturing processes and process controls for the drug product and the lipid components are required. Extensive characterization of the composition, variability, and stability of the liposomal component is required.

## Human pharmacokinetics: Bioavailability and bioequivalence

When considering the complexity and drug release patterns compared with other LPMs or non-LPM products, FDA noted that measuring the concentration of total drug in plasma is too simple and is insufficient to indicate bioequivalence. Hence, the measurement of total drug concentration at the target site must be determined. A pre-ANDA meeting is recommended for ANDA applicants prior to submission, to determine an appropriate method for assessing the bioavailability of drugs at the site of action. In addition, NDA applicants should consult with the appropriate CDER review division. The guidance indicates that a proposed innovative product and an approved non-LPM product should be compared if both of the products share the same formulation ingredient and administration route, to evaluate any differences in ADME studies. In addition, a mass balance study with radio-labeled drugs in both LPM and non-LPM products could be useful to identify any differences in drug distribution behavior in the organs of interest. A single-dose PK study should be performed in an appropriate patient population to compare the LPM and non-LPM products. The guidance indicates that release studies using the same drug must be performed for LPM and non-LPM products to determine if they exhibit similar release behavior. FDA encourages attempts to perform *in vitro*/*in vivo* correlation studies. Potential interactions between LPMs and serum proteins should be investigated to determine the significance of “dose-dumping” of the drug *in vivo*. Reports of prior studies of LPM–protein interactions may be sufficient for new LPM drug products and can be used if the physiochemical properties of both LPM drug products are similar and their lipid composition and active ingredients are the same. Regarding labeling, FDA recommends including a cautionary statement in the product description section stating that some active ingredients may exhibit a potential difference in behavior between the proposed innovative product and other LPM and non-LPM products. However, a therapeutically equivalent LPM drug product as determined by FDA does not need a cautionary label.

## FDA guidance for industry on drug or biological products that contain nanomedicine

FDA provides guidance on the development of human drug products that are also biological products and which contain NM in the finished dosage form (FDA-2017-D-0759:2022). To date there is no regulatory definition of “nanotechnology”, “nanomaterial,” “nanoscale”, or any other related terms. FDA considers its products or materials with with any internal or external structures on the nanoscale dimension (1–100 nm) as nanomaterials. This guidance does not apply to biological products composed of proteins, cells, viruses, nucleic acids, or other biological materials that occur naturally at particle sizes ranging up to 1 μm (1,000 nm), unless the material exhibits dimension-dependent properties or components up to 1 μm are also present in the product.

The description of the NM in a drug product must be included in any premarket application and must include size, charge, morphology, and complexation. In addition, a description of the functionality of the NM must be included. Several attributes of the NM in the drug product must be included in the premarket application, such as chemical composition, average particle size, assay of drug substances, *in vitro* release profile etc. For the characterization of NM, standardized methods exist, and the applicants must justify the use of any standardized methods used for the tested product. Dissolution/*in vitro* drug release methods with detailed descriptions and various parameters involved in the study must be provided in the submission. Drug release should reach a plateau or achieve at least 85% of drug release. Incomplete drug release profiles must be accompanied in the premarket application by sufficient data to explain the incomplete release. FDA encourages applicants to devise their own drug release methods and they must consult with FDA regarding repeatability, scientific relevance, etc. Batch to batch variations in bulk-scale production is an issue, because even a slight change in an NM’s properties could have a serious impact on its *in vivo* behavior. Improving the manufacturing process with appropriate controls to prevent cross-contamination is necessary. In addition, if the produced NM lacks surface coating or is missing a component this could be considered an impurity and must be quantified. NMs used as an excipient are expected to improve the desired attributes of a product without exerting any therapeutic functions. Such NM excipients must be characterized based on their function and intended use. This characterization should include relevant controls, test methods, acceptance criteria, and a description of the source of materials. The study of nanomaterials’ stability in products should involve an evaluation of any physiochemical changes during handling and storage. Stability issues, such as a change in NM size, morphology, charge, release rate of drug, drug leakage during storage, or degradation, must be considered. If dilution is necessary for drug products before usage, the dilution medium may affect the colloidal stability. So, FDA recommends in-use stability studies at clinically relevant concentrations, which should evaluate any reactions of the NM with the sample container walls or the administration or delivery device.

International Council for Harmonisation guidance for the nonclinical safety of drug products is generally applicable to drug products containing NM. NMs present in the drug products or as a carrier of a drug must be evaluated for their ADME parameters, and their impact on safety should be determined. For *in vivo* biodistribution, NMs should be labeled to specify their accumulation in organs. Administration route-specific issues must be taken into account when evaluating the safety of drug products containing NM. If an NM is included in a previously approved drug product, ADME and a bridging toxicology evaluation is appropriate to allow reliance on FDA’s findings of efficacy and safety of previously approved drug product.

## Nanomedicine design challenges

The physiochemical properties and stability of NM are difficult to control, and the lack of comprehensive standards may be the reason for the challenges in clinical development. Multifunctional NM, which has more complicated design and synthesis, may be more challenging than for other INDs; however, it can also provide more information for diagnostics and be a more effective treatment than a single-function NM. We need to determine whether the benefits of multifunctional NM can allow us to ignore the challenges in clinical development. Multistep NM, which encapsulates different drugs in different nanocarriers, can also achieve disease treatment that can be accomplished by multifunctional NMs. For instance, NM separately encapsulating Bcl-2 siRNA and Dox could overcome the problem of multiple drug resistance (Kim et al., 2014). Simplification through nanocarrier design can enable more nanocarriers to achieve clinical benefits for humanity. However, multifunctional NMs are still a necessity in certain situations. Simultaneously treating patients and monitoring NP targeting through the use of theranostic nanocarriers can provide important feedback to physicians, such as whether a tumor’s failure to respond arises from drug resistance or insufficient drug delivery. Multifunctional NM is also essential for some particular conditions, such as SCI. Following an SCI, various drugs must be injected into the injured area at different stages to avoid massive necrosis of the spinal nerves and to help repair the spinal nerves. Multifunctional NM, which can release drugs at different timepoints, can prevent unnecessary side effects that arise from repeated drug injections in the injured area.

## Conclusion

Recent developments in NM have yielded highly promising results for the clinical application of NPs in the treatment of disease, especially cancer. Multifunctional theranostic carriers have demonstrated that one particle need not only be used for therapeutic or diagnostic applications but can instead be used to provide both diagnostic information and feedback from the site of therapeutic intervention. Furthermore, multitherapy NPs can realize the effective treatment of disease that single-therapy NPs cannot achieve. Due to the lack of regulatory experience with NM, however, it is more difficult to conduct clinical trials for multifunctional NM, which has a complicated design. Compared with multifunctional NM, multistep NM has a simpler design and corresponds to the requirements of clinical needs. However, multifunctional NM is also important in certain diseases, such as SCI. Fortunately, ISO and ASTM International have published several standardized documents related to NM. In the future, the clinical importance of NM is expected to increase markedly with the development of relevant standards and its necessity for treating certain diseases.
